# Venesection in Uræmia

**Published:** 1910-03-19

**Authors:** 


### Medicine.

### VENESECTION IN URÆMIA.

The mistrust of venesection that arose in consequence of its indiscriminate use and abuse in former years has latterly decreased to some extent, if, indeed, it has not disappeared, and there should now be no doubt that venesection under certain circumstances is distinctly indicated. Besides its use in relieving the right side of the heart in mitral stenosis or pneumonia, it has been found particularly beneficial in the treatment of uræmia when the kidneys are acutely inflamed. It sometimes acts almost miraculously in checking uræmic convulsions, and occasionally may do so almost instantaneously.

As to the manner in which venesection acts in patients suffering from uræmic poisoning opinions are divided. The view most generally accepted is that a considerable amount of poisonous substance is removed at the same time as the blood, and that a favourable result depends upon the amount of poison thus removed. So simple an explanation, however, scarcely accounts for the rapidity with which the venesection sometimes acts. The amount of blood left in the body is not materially lessened by the venesection, and although the absolute quantity of poison circulating in the blood must be slightly less than before, it is true nevertheless that the concentration of this poison in the blood bathing the tissues would not be very materially diminished, or at least could scarcely be supposed to be diminished instantaneously. Indeed, if the amount of blood removed is, as some argue, replaced almost at once by fluids withdrawn from the tissues, one would suppose that the concentration of the poison in the organs of the body would be actually increased, and it is on account of such possible increase that most authorities nowadays advocate the infusion of saline solution at the same time that venesection is performed in these cases. Nevertheless, experience shows that venesection alone saves nearly as many cases of acute uræmia as venesection plus infusion, whilst infusion without venesection is far less beneficial than might have been expected. Indeed, so remarkable may be the improvement in the uræmic symptoms that some authorities think that the actual removal of poison in the blood withdrawn must be but a minor factor, and it has been suggested that the venesection really acts by relieving some vaso-motor spasm of the arteries, either in the kidneys or elsewhere.

It has been suggested by Stursberg that if uræmia be due to a poison in the blood and in the tissues, or, if Soelbeer's view is right, only in the tissues, particularly in the central nervous system, then one would expect uræmic symptoms as soon as the concentration of the poison in the organs has reached a certain point. If now the venesection causes a transit of fluid from the tissues to the vascular system, then one might expect a certain amount of the poison to be suddenly withdrawn from the tissues at the same time, and in that case it would be easy to understand the sudden disappearance of the severer symptoms. It would not be so much a dilution of the blood, or the direct abstraction of poison from the blood as a removal of the excess of poison from the tissues into the blood, and this would explain how it is that venesection, even without infusion, may do so much good. If this hypothesis is to be proved, or even to be rendered probable, it would be necessary to demonstrate that the fluid returning to the blood from the tissues actually is rich in such substances as may be regarded as likely to produce uræmia, particularly those which are in some way related to the constituents of the urine.

This proof is necessarily very difficult to obtain in the case of most of the urinary substances, and it is impossible in connection with the unknown poison of uræmia; but something may perhaps be learnt in this respect from the behaviour of some of the pigmentary substances which the kidneys excrete. Nussbaum some time ago experimented with one such substance, namely sulphindigotate of sodium, and although his original work did not directly bear upon uræmia, the results he found were as follows: If a solution of sulphindigotate is injected intravenously into a dog or a rabbit, and if blood is then obtained by venesection 25 minutes later, no spectrum absorption band of the indigotate is to be seen in the 60 times diluted liquor sanguinis, although this band is easily seen in an ordinary saturated solution of the salt diluted 1,500 times. When the animal was bled completely to death, however, then the absorption band became visible in the last few cubic centimetres removed when these were diluted 60 times as before. It would seem to follow from these experiments that the colour material stored up in the tissues returned to the circulation as the result of excessive venesection. At any rate, whether this conclusion is warrantable or not, the experiments caused Stursberg to carry out some further investigations upon this point. He used a special apparatus for determining the concentration of the pigment in the blood, and he employed rabbits into which he injected definite quantities of sodium sulphindigotate solution intravenously. Blood was withdrawn from a vein after 20, 25, and 30 minutes, each specimen of blood was defibrinated and the serum tested with an instrument for determining the pigment. He found that under certain circumstances a considerable outflow of urinary substances may take place from the overladen tissues into the blood stream as the result of a simple venesection without infusion. If this view be true, then it agrees with the fact that has been observed clinically again and again that venesection may relieve uræmia without any simultaneous infusion, seeing that the relief to the uræmia that venesection produces depends not upon the dilution of the poison in the blood itself, nor upon the absolute amount of poison removed in the blood taken from the vein, but upon the fact that the amount of poison in the saturated tissues is rapidly reduced to a point which is consistent with the continuance of life without uræmic symptoms. The dilution of the blood that a simultaneous infusion with normal saline solution is likely to bring about will naturally be of further service in the treatment of the case, but mere transfusion without venesection would not seem to be the quickest means of diminishing the concentration of the poison in the tissues themselves. In other words, if the choice lies between venesection and transfusion in a given case, venesection should be chosen first; if circumstances allow of both being performed, then venesection should be followed by infusion.

